# The Olive Phenolic *S*-(-)-Hydroxyoleocanthal Attenuates Neuroendocrine Prostate Cancer via Modulation of EPHA3-Centered Oncogenic Network

**DOI:** 10.3390/cancers18010026

**Published:** 2025-12-21

**Authors:** Md Towhidul Islam Tarun, Hassan Y. Ebrahim, Khalid A. El Sayed

**Affiliations:** 1 School of Basic Pharmaceutical and Toxicological Sciences, College of Pharmacy, University of Louisiana at Monroe, 1800 Bienville Drive, Monroe, LA 71201, USA; tarunmt@warhawks.ulm.edu (M.T.I.T.); hebrahim@ulm.vcom.edu (H.Y.E.); 2 Department of Biomedical Sciences, Discipline of Pharmacology, Edward Via College of Osteopathic Medicine, Monroe, LA 71201, USA

**Keywords:** *S*-(-)-hydroxyoleocanthal, neuroendocrine prostate cancer, olive phenolics, progression, recurrence, RNA-sequencing, EPHA3–BRN2–EZH2–ASCL1–DLL3–SYP–CHGA axis

## Abstract

**Background/Objectives.** Prostate cancer (PCa) is the second leading cause of cancer-related mortality among men in the United States. Treatment with second-generation androgen receptor (AR) inhibitors, such as enzalutamide, can trigger lineage plasticity, promoting the transdifferentiation of PCa cells into an AR-independent, poorly differentiated neuroendocrine phenotype (NEPC). The receptor tyrosine kinase EPHA3 is a critical driver for NEPC. It is overexpressed in PCa, particularly in androgen-independent and neuroendocrine subtypes. EPHA3 activates c-Myc signaling to enhance EZH2 expression, promoting histone H3K27 trimethylation. The neural transcription factor BRN2 functions upstream of both EZH2 and ASCL1. The latter regulates the Notch pathway ligand DLL3, thereby orchestrating neuroendocrine differentiation. Elevated expression of classical neuroendocrine markers CHGA and SYP is characteristic of the NEPC phenotype. This study reports the novel usage of the olive phenolic *S*-(-)-hydroxyoleocanthal (HOC, oleacein) to effectively control NEPC by targeting the EPHA3–BRN2–EZH2–ASCL1–DLL3–SYP–CHGA oncogenic network. **Methods.** Cell viability assays were conducted to assess in vitro effects. To model NEPC progression and recurrence, NCI-H660-Luc cells were xenografted into male athymic nude mice. RNA-sequencing was performed to compare the differentially expressed genes between placebo control and treated tumors. **Results.** HOC significantly attenuated the proliferation of NEPC NCI-H660 cells in vitro. Daily oral administration of HOC at 10 mg/kg body weight markedly suppressed the progression of NEPC NCI-H660-Luc tumors. Continued HOC treatments after surgical excision of the primary tumors substantially reduced locoregional recurrence. HOC significantly downregulated the expression of EPHA3, BRN2, EZH2, ASCL1, DLL3, SYP, and CHGA in treated primary and recurrence tumors versus placebo control. **Conclusions.** These findings establish HOC as a multifaceted therapeutic entity capable of disrupting key NEPC oncogenic networks, highlighting its potential as a novel lead intervention for aggressive NEPC.

## Simple Summary

*S*-(-)-Hydroxyoleocanthal (HOC, oleacein), a natural phenolic derived from extra-virgin olive oil, demonstrates potent inhibitory effects against neuroendocrine prostate cancer (NEPC), the most aggressive subtype of prostate cancer (PCa). Daily oral administration of HOC in a nude mouse NEPC xenograft model markedly suppressed the tumor expression of *EPHA3* and *BRN2*, along with their downstream effectors *EZH2*, *ASCL1*, and *DLL3* and neuroendocrine markers *SYP* and *CHGA*. Disruption of this signaling axis impaired NEPC progression and recurrence. These findings suggest that HOC targets critical molecular pathways driving PCa lineage plasticity and aggressiveness, supporting its potential as a promising nutraceutical lead candidate for NEPC control.

## 1. Introduction

Prostate cancer (PCa) is the most commonly diagnosed malignancy and the second leading cause of cancer-related mortality among men in the United States. In 2025, PCa is projected to account for approximately 30% of all male cancers, with an estimated 313,780 new cases and 35,770 deaths [1,2]. Nearly one in every eight men will develop PCa over their lifetime, and approximately 80% of PCa-related deaths occur in men aged ≥ 70 years [1,2]. The androgen receptor (AR) plays a central role in the initiation and progression of PCa, promoting tumor cell proliferation and survival through androgen-dependent signaling. Despite initial responsiveness to androgen deprivation therapy (ADT), the disease often progresses to castration-resistant prostate cancer (CRPC), an advanced stage associated with poor prognosis and limited therapeutic options [3]. Although de novo neuroendocrine prostate cancer (NEPC) is rare (≤1% of cases), treatment-emergent NEPC (t-NEPC) develops in approximately 20–25% of patients with metastatic CRPC following a prolonged hormonal therapy course [4]. NEPC has some degree of molecular and pathological similarity to small cell lung cancer (SCLC). The platinum-based chemotherapies are commonly employed interventions for NEPC; however, their clinical benefits are limited [3,4].

EPHA3 (erythropoietin-producing hepatocellular carcinoma cell surface type A receptor 3) is a member of the EPH receptor tyrosine kinase (RTK) family, which is overexpressed aberrantly in several malignancies, including PCa [5,6]. EPHA3 contributes to tumorigenesis, progression, and metastatic potential [5,6]. Elevated EPHA3 expression correlates with poor clinical outcomes and reduced overall survival in PCa patients [7]. Notably, EPHA3 is upregulated in androgen-independent and CRPC cell lines [8,9]. Mechanistically, EPHA3 activates c-Myc signaling, leading to increased expression and activity of the lysine methyltransferase enhancer of zeste homolog 2 (EZH2) [5,6]. EZH2 subsequently promotes histone H3K27 trimethylation (H3K27me3)-mediated silencing of PTEN, driving oncogenic activation of the Akt and epithelial–mesenchymal transition (EMT) pathways [5].

Delta-like ligand 3 (DLL3), a noncanonical ligand of the Notch pathway, has emerged as a key driver of tumor progression and poor clinical prognosis in NEPC [10,11]. DLL3 is transcriptionally regulated by achaete-scute homolog 1 (ASCL1), a lineage-defining transcription factor that governs neuroendocrine differentiation (NED). Aberrant cellular surface expression of DLL3 is observed in the majority (~76.6%) of NEPCs, underscoring its potential as diagnostic and therapeutic molecular target [12].

ASCL1 and EZH2 exhibit a complex crosstalk coordination in NEPC pathogenesis. ASCL1 promotes NED and is markedly upregulated in NEPC [13], while EZH2, an epigenetic silencer, is frequently overexpressed, driving lineage plasticity [14]. Studies demonstrate a strong positive correlation between ASCL1 and EZH2 expression, where ASCL1 modulates EZH2 activity, and EZH2 reciprocally regulates ASCL1 expression and neuronal gene networks, establishing a feed-forward regulatory loop that sustains the neuroendocrine phenotype [14,15].

The neural transcription factor BRN2 (POU3F2) is another critical regulator of NEPC development and progression [16]. BRN2 expression is markedly dysregulated in NEPC and metastatic CRPC, particularly in tumors characterized by diminished AR signaling [16]. BRN2 directly induces ASCL1 expression and reinforces the neuroendocrine transcriptional program [17]. Furthermore, BRN2 regulates EZH2 expression, thereby influencing the epigenetic landscape and promoting NEPC lineage reprogramming [18]. Targeting BRN2 suppresses proliferation in NEPC models with concurrent downregulation of EZH2 and other neuroendocrine markers including synaptophysin (SYP) and chromogranin A (CHGA) [17,19,20].

Epidemiological studies have shown a strong correlation between the adherence to Mediterranean diets rich in extra-virgin olive oil (EVOO) and reduced incidence of multiple cancer types [21]. EVOO contains minor unique bioactive phenolic secoiridoids, notably *S*-(-)-oleocanthal (OC) and *S*-(-)-hydroxyoleocanthal (oleacein; HOC). OC and HOC exerted documented antioxidant, anti-inflammatory, and anticancer effects [22]. OC studied more comprehensively for oncological suppressive effects, exhibiting broad anticancer activity by targeting oncogenic mediators including c-MET, SMYD2, and EZH2, thereby suppressing tumor proliferation, invasion, progression, and recurrence in multiple cancer types, including breast, colorectal, and metastatic CRPC models [23,24,25]. Replacement of the OC phenolic alcohol tyrosol with hydroxytyrosol (HT) afforded HOC, a dialdehydic derivative of oleuropein aglycone, with an *o*-catechol aromatic ring system. HOC exhibited potent antioxidant, anti-inflammatory, anti-proliferative, and antimicrobial activities [26]. HOC inhibited melanoma and neuroblastoma cells proliferation by inducing cell cycle arrest and apoptosis through modulation of oncogenic genes, activation of p53 and Bax, suppression of Bcl-2, and inhibition of STAT3 phosphorylation, highlighting its potential as an anticancer entity [27,28]. Additionally, HOC effectively targeted tumor-initiating cancer stem cells across genetically diverse tumors [29] and exhibited angiopreventive properties [30].

Despite growing evidence of the anticancer potential of EVOO-derived phenolics, there are currently no reports describing natural products with NEPC-suppressive activity. Based on its multitargeted oncogenic modulation and potent activity of OC against mCRPC [24], the structurally related HOC is hypothesized as a prospective suppressor for PCa by disrupting key molecular signaling networks. This study presents the novel in vivo lead validation of HOC anti-NEPC activity, providing molecular mechanistic insights and highlighting HOC potential as a novel lead intervention appropriate for effective NEPC management.

## 2. Materials and Methods

### 2.1. Chemicals and Reagents

All chemicals utilized in this study were obtained from Avantor Science Central (Allentown, PA, USA), unless otherwise specified. HOC was isolated from the Greek EVOO Governor (Corfu, Greece) through a liquid–liquid extraction process, followed by adsorption onto SP70 entrapment resin [31]. After displacement of residual water, HOC-rich fraction was eluted with acetone and subsequently purified on Sephadex LH20 using CH_2_Cl_2_ with increasing concentrations of EtOAc as a gradient-eluted mobile phase. The final purified HOC exhibited ≥99% purity, confirmed by q^1^H NMR analysis [31].

### 2.2. Compound Preparation and Stock Solution

HOC was dissolved in dimethyl sulfoxide (DMSO) to provide a stock solution of 25 mM, which is used to prepare different concentrations of HOC treatment media. DMSO final concentration was maintained fixed in all treatment groups within all given experiments and never exceeded 0.5% of each.

### 2.3. Cell Lines and Culture Conditions

The human PCa cell lines lymph node carcinoma of the prostate cell line LNCaP (androgen-dependent), Duke University 145, DU145, a human prostate carcinoma cell line derived from a central nervous system metastasis and prostate carcinoma 3-metastatic cell line PC-3M (type-I androgen-independent), and prostate carcinoma 3 cell line PC-3 (castration-resistant) were obtained from the American Type Culture Collection (ATCC, Rockville, MD, USA). The metastatic castration-resistant PCa cell line Case Western reserve recurrent 1 carcinoma (fibroblast-free) CWR-R1ca cell line was obtained from Millipore Sigma (Burlington, MA, USA). Cells were cultured in RPMI-1640 medium supplemented with 10% fetal bovine serum (FBS), 2.5 µg/mL amphotericin B, 100 µg/mL streptomycin, and 100 IU/mL penicillin G, and maintained at 37 °C in a humidified incubator with 5% CO_2_. Subculturing was performed at 70–80% confluency. Cells were washed with Ca^2+^-free phosphate-buffered saline (PBS) and detached using 0.05% trypsin–0.02% ethylenediaminetetraacetic acid (EDTA) for up to 5 min at 37 °C. Trypsinization was terminated by addition of complete growth medium, and cells were collected by centrifugation and resuspended in fresh medium.

The human NEPC cell line National Cancer Institute-human 660, NCI-H660, was obtained from the American Type Culture Collection (ATCC, Rockville, MD, USA). Cells were maintained in ATCC-formulated RPMI-1640 medium supplemented with 0.005 mg/mL insulin, 0.01 mg/mL transferrin, 30 nM sodium selenite, 10 nM hydrocortisone, 10 nM 17β-estradiol, an additional 2 mM L-glutamine (final concentration 4 mM), and 5% FBS. Cultures were maintained at 37 °C in a humidified incubator with 5% CO_2_. Cell density was monitored regularly, and medium was replaced every 2–3 days. For subculturing, flasks were gently agitated to dislodge aggregated clusters, and passaging was performed when multiple healthy clusters were observed to ensure continued proliferation and viability.

### 2.4. Cells Viability Assay

LNCaP, DU145, PC-3, PC-3M, and CWR-R1ca cells were seeded in 96-well plates at a density of 5000 cells per well in RPMI-1640 medium supplemented with 10% FBS and allowed to attach overnight. Cells were then treated with varying concentrations of HOC reconstituted in 25 mM DMSO and vehicle control (VC) for 72 h. At the end of the treatment, cell viability was assessed using the 3-(4,5-dimethylthiazolyl-2)-2,5-diphenyltetrazolium bromide (MTT, VWR International, Suwanee, GA, USA) assay, following previously described protocols [24,25].

The non-adherent NCI-H660 cells were seeded at 5000 cells per well in 96-well plates and treated with HOC in a complete RPMI-1640 medium containing 5% FBS. Cells were incubated for 5 days at 37 °C in a humidified atmosphere with 5% CO_2_. Subsequently, 20 µL of MTS reagent was added to each well, and plates were incubated for 3–4 h to allow color development. Absorbance was measured at 490 nm using a microplate reader (BioTek, Winooski, VT, USA). Values were normalized to vehicle-treated controls, and data were analyzed to determine the effects of HOC on cells’ viability.

### 2.5. Lentivirus-Aided Luciferase Labeling of NCI-H660 Cells

NCI-H660 cells were seeded in 12-well plates and cultured until reaching 60–70% confluency. Lentiviral particles encoding the luciferase gene (Kerafast, Boston, MA, USA) were prepared by mixing with Opti-MEM reduced-serum medium (1.5 µL per 100 µL) on ice. Culture medium was aspirated, cells were washed with PBS, and 100 µL of Opti-MEM with or without viral particles was added to each well. Cells were incubated for 4–6 h at 37 °C, after which the transfection medium was replaced with complete serum-containing medium, and cells were cultured for several days. Stable luciferase-expressing cells were selected by adding puromycin (15 µg/mL; Santa Cruz Biotechnology, Dallas, TX, USA) to the culture medium, which was refreshed every 2 days to eliminate non-transduced cells. Luciferase activity was evaluated by adding 20 µL of 50 mM D-luciferin potassium salt bioluminescent substrate (PerkinElmer, Waltham, MA, USA) in PBS to each well, followed by incubation for 6 min at room temperature. Bioluminescence was confirmed using the IVIS imaging system (PerkinElmer, Waltham, MA, USA), ensuring successful luciferase tagging [24,25].

### 2.6. Western Blot Analysis

Approximately 1 × 10^6^ NCI-H660 cells were treated with HOC for 72 h, and cell lysates were collected as previously described [24,25]. Protein bands were detected using the Chemi-Doc XRS chemiluminescent imaging system and quantified with Image Lab software (Bio-Rad v5.2.1, Hercules, CA, USA). β-Tubulin was used as a loading control. All experiments were performed in triplicates and representative images are presented in the result figures.

### 2.7. Animal Models and Treatments

Male athymic nude mice (Foxn1^nu^/Foxn^1+^, 4–5 weeks old) were obtained from Envigo (Indianapolis, IN, USA) and housed at the University of Louisiana at Monroe (ULM) vivarium. Mice were acclimated for one week under sterile conditions in ventilated, filter-top cages with AlphaDri bedding. The vivarium was maintained at 24 ± 2 °C, 50–65% relative humidity, with a 12 h light/dark cycle. Cages were cleaned, and bedding was replaced twice weekly. Mice had access to sterile water and pelleted rodent chow containing 5% fat (Cat #7012, Envigo-Teklad, Madison, WI, USA). All animal procedures were conducted in accordance with NIH guidelines and approved by the ULM Institutional Animal Care and Use Committee (IACUC) under protocol numbers 23-MAR-KES-02. Clinical health parameters, including food and water consumption, defecation, urination, physical activity, and body weight, were monitored daily throughout the study.

### 2.8. Nude Mouse Xenograft Model

Approximately 2 × 10^6^ NCI-H660-Luc cells suspended in 100 µL of Matrigel were subcutaneously injected into the right flank of each male nude mouse. Tumor growth was monitored daily, and tumor volume (V) was calculated using the formula V = (L/2) × W^2^, where L and W represent the tumor length and width, respectively. The study design consisted of two experimental phases—tumor progression and tumor recurrence. Once tumors reached ~50 mm^3^, mice were randomly assigned into two groups, *n* = 5 each: (i) placebo control and (ii) 10 mg/kg daily oral HOC treatment. The accurately weighed HOC treatment was formulated in de-phenolized EVOO (dpEVOO), which served as the optimal vehicle for HOC oral systemic delivery [31]. The final dosing volume was adjusted based on each mouse’s body weight. Oral dosing was administered using 2 mm diameter gavage tubes with stainless-steel bite protectors (23-gauge, 3.81 cm length) for 51 consecutive days. Tumor volume was measured every other day using a digital caliper. At the end of the progression phase, mice were anesthetized with isoflurane, and primary tumors were surgically excised under aseptic conditions. Post-surgical wounds were monitored to ensure proper healing, and mice were observed closely for 24 h to prevent infection or distress. The following day, oral treatments resumed according to their originally assigned groups (placebo or 10 mg/kg HOC) and continued for an additional 75 days to assess tumor recurrence. At study completion, mice were anesthetized with isoflurane and euthanized. Tumors and major organs were collected, weighed, and stored at −80 °C for subsequent total protein extraction and Western blot analyses.

### 2.9. RNA Extraction

Excised tumors were snap-frozen and stored at −80 °C until processing. Approximately 50 mg of tumor tissue was placed in 1 mL of TRIzol reagent (Cat. #15596026, ThermoFisher, Waltham, MA, USA) in an RNase-free tube and incubated for 30 min. Tumors were homogenized using a tissue homogenizer at medium speed for 30 s, repeated three times with 1 min intervals on ice between each round to ensure complete lysis. RNA extraction, including phase separation, precipitation, washing, and resuspension, was performed following the manufacturer’s instructions. RNA concentration and purity were determined using a NanoDrop spectrophotometer (ThermoFisher, Waltham, MA, USA), and samples were stored at −80 °C for subsequent analyses.

### 2.10. RNA Sequencing and Data Analysis

RNA sequencing was performed at the University of Kansas Medical Center Genomics Core using a strand-specific, 100-cycle paired-end configuration on the Illumina NovaSeq 6000 platform (Illumina, San Diego, CA, USA). NCI-H660-Luc samples treated with HOC, or vehicle control were multiplexed across two lanes of a flow cell, generating 40.0–48.7 million reads per sample. Read quality was assessed using FastQC software v0.12.0 [32], with an average Phred score > 30 across all samples, and no adapter sequences required trimming. Reads were aligned to a combined human (GRCh38) and mouse (GRCm39) reference genome using STAR (v2.7.11b2) [33], achieving an average mapping rate of 91%, of which 80.4% were uniquely aligned, yielding 36.0–42.9 million uniquely mapped reads per sample. Transcript abundance was quantified with FeatureCounts v2.07 [34], and differential gene expression analysis was performed using DESeq2 v1.48.2 [35]. *p*-values were adjusted for multiple testing using the Benjamini–Hochberg method to control the false discovery rate (FDR) for each [36].

### 2.11. Protein–Protein Interactions

Differentially expressed genes (DEGs) were analyzed using STRING with a confidence threshold of 0.4. This score, ranging from 0 to 1, reflects the predicted likelihood of a valid interaction, with 1 indicating the highest confidence. STRING evaluates interactions based on multiple lines of evidence, including genomic context, gene co-expression, experimental data, molecular pathways, automated text mining, and curated databases.

### 2.12. Statistics

Data were analyzed using GraphPad Prism version 8.4.3 (La Jolla, CA, USA). Continuous variables are presented as mean ± standard deviation (SD). Statistical comparisons were performed using Student’s *t*-tests, as appropriate. Statistical significance was defined as * *p* < 0.05, ** *p* < 0.01, *** *p* < 0.001, and **** *p* < 0.0001, and ns means non-significant.

## 3. Results

### 3.1. Antiproliferative Activity of HOC Against Diverse PCa Cell Lines

**Figure 1:**
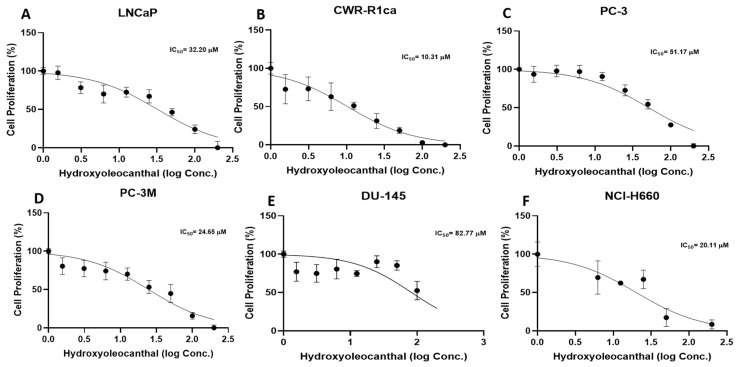
Effects of HOC on the viability of diverse PCa cell subtypes. Antiproliferative effects of HOC against the human PCa cell lines LNCaP, CWR-R1ca, PC-3, PC-3M, DU-145, and 660 NCI-H660.

HOC was evaluated for its in vitro suppressive effects on the viability of a diverse panel of human PCa cell lines, including the androgen-dependent LNCaP, androgen-independent PC-3M and DU-145, CRPC CWR-R1ca and PC-3, as well as the NEPC cell line NCI-H660 (Figure 1). The calculated IC_50_ values for LNCaP, CWR-R1ca, PC-3, DU-145, PC-3M, and NCI-H660 were 32.2 μM, 10.3 μM, 51.2 μM, 82.8 μM, 24.7 μM, and 20.1 μM, respectively. Notably, HOC exhibited the strongest cytotoxic activity against the castration-resistant CWR-R1ca and the NEPC NCI-H660 cell lines, suggesting its enhanced efficacy against more aggressive and therapy-resistant PCa subtypes.

### 3.2. HOC Suppressed the NEPC Progression in a Nude-Mouse NCI-H660-Luc Cells-Developed Xenograft Model

**Figure 2:**
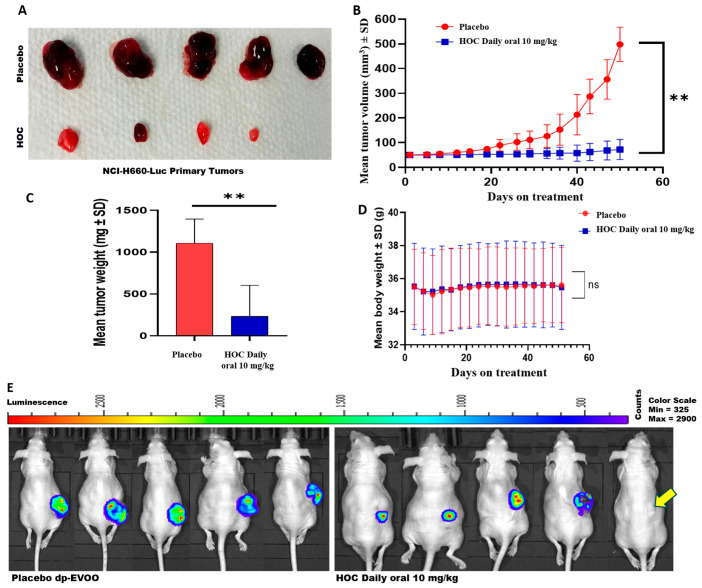
Effects of HOC on the progression of NEPC NCI-H660-Luc tumors in a nude mouse xenograft model. (**A**) Representative images of tumors collected from HOC-treated and placebo control groups following primary tumor excision. (**B**) Longitudinal monitoring of NCI-H660-Luc tumor volume over the study course. (**C**) Comparison of final tumor weights between HOC-treated and placebo control mice. (**D**) Average body weight profiles of NCI-H660-Luc tumor-bearing nude mice over the study course. (**E**) Comparison of the whole-body bioluminescent imaging of placebo versus HOC-treated male nude mice at the end of the progression study phase before primary tumors surgical excision. Yellow arrow defines the HOC-treated mouse with full tumor regression over progression phase. Results are expressed as mean ± SD. ** *p* < 0.01.

Daily oral administration of HOC at 10 mg/kg body weight for 51 days in NCI-H660-Luc cell-xenografted male nude mice led to a substantial reduction in tumor burden compared to the placebo dp-EVOO-treated group (Figure 2A–E). Specifically, HOC oral treatments suppressed NEPC NCI-H660 cell tumor volume and weight by 85.5% and 79.1%, respectively, relative to the placebo control-treated animals (Figure 2A–C). Remarkably, complete tumor regression was observed in one out of the five HOC-treated mice, showing no evidence of bioluminescent tumors entirely prior to primary tumor surgical excision (Figure 2E). This is translated to a 20% full recovery of this highly aggressive PCa subtype. The remaining HOC-treated four mice exhibited an average of approximately 85.5% reduction in tumor volume compared to the placebo group, underscoring the robust antitumor in vivo efficacy of HOC.

Throughout the treatment course, no significant differences in animals’ body weight were noted between the HOC and placebo-treated groups, indicating that HOC was well tolerated and did not cause any overt toxicity (Figure 2D).

### 3.3. HOC Suppressed the Locoregional and Distant Recurrences After the NCI-H660-Luc Primary Tumors Surgical Excision in a Nude-Mouse Xenograft Model

**Figure 3:**
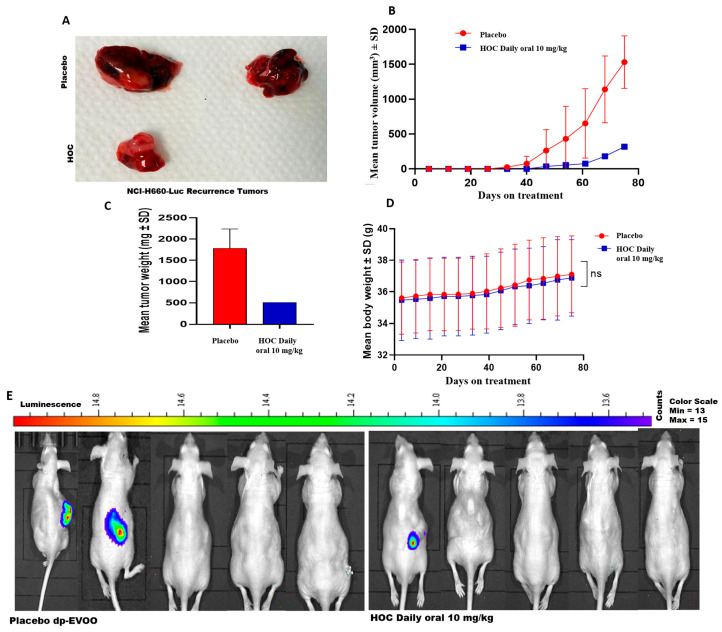
The suppressive effects of HOC on NEPC tumor recurrence. Daily oral administration of HOC (10 mg/kg) for 75 days after surgical removal of primary tumors significantly reduced locoregional recurrence of NCI-H660-Luc tumors in nude mice. (**A**) Representative images of tumors collected from HOC-treated and placebo control groups after recurrence phase. (**B**) NCI-H660-Luc recurrence tumor volume monitoring throughout the recurrence study period. (**C**) Comparison of the final tumor weights of HOC-treated mice versus placebo controls during the recurrence phase. (**D**) Mean body weight trends of NCI-H660-Luc tumor-bearing nude mice throughout the treatment period in the recurrence phase. (**E**) Comparison of the whole-body bioluminescent imaging of the locoregional tumor recurrence in male nude mice treated with HOC versus placebo control at the end of the NCI-H660-Luc tumor recurrence study phase. Statistical analysis was performed using a student’s *t*-test, where ns denotes no significant difference.

The NCI-H660-Luc xenografted male athymic nude mice were subjected to primary tumor surgical excision followed by continued daily oral 10 mg/kg HOC or placebo dp-EVOO control treatments. This study recurrence phase was monitored for 75 days post-surgery. HOC treatments markedly suppressed tumor locoregional recurrence, evidenced by the 79.3% and 71.5% reductions in recurrence tumors volume and weight, respectively, compared to the placebo-treated control (Figure 3A–C). Tumor recurrence incidence was also notably reduced in the HOC-treated group. Among the placebo-treated mice, 2 out of 5 animals developed locoregional recurrences by the study end, whereas only 1 out of 5 HOC-treated mice showed locoregional recurrence. The HOC treatment group included the single mouse that exhibited complete tumor regression during the progression phase, which did not develop tumor recurrence over the 75-day recurrence phase. Luciferin-aided bioluminescence imaging examination of the collected mouse organs including kidney, spleen, liver, heart, lung, brain, and intestine spotted only one distant tumor recurrence in the brain of a placebo control-treated mouse (Supplementary Figure S1). None of HOC-treated mouse organs showed any active bioluminescent tumors. Moreover, there were no changes in the animals’ body weight during the recurrence phase in either of the study groups, suggesting HOC’s preliminary safety profile at the used therapeutic dose (Figure 3D). Gross morphological examination further supported the anti-angiogenic effects of HOC. The recurrent tumor from HOC-treated mice displayed visibly reduced vascularity compared to the placebo control group, suggesting the potential of HOC to abort the NEPC tumor ability to recruit vascular endothelial cells (Figure 3A). HOC treatments also delayed tumor recurrence onset further supports HOC’s efficacy. In the placebo-treated group, locoregional recurrence initiated between days 33 and 40 post-surgery, whereas in HOC-treated mice, recurrence began on day 47, corresponding to a 14-day delay in recurrence initiation (Figure 3B). The limited number of recurred tumors (2 in placebo control and one in HOC treatment) precluded the statistical significance calculation (Figure 3B,C).

### 3.4. EPHA3 Differential Regulation in NEPC Model

**Figure 4:**
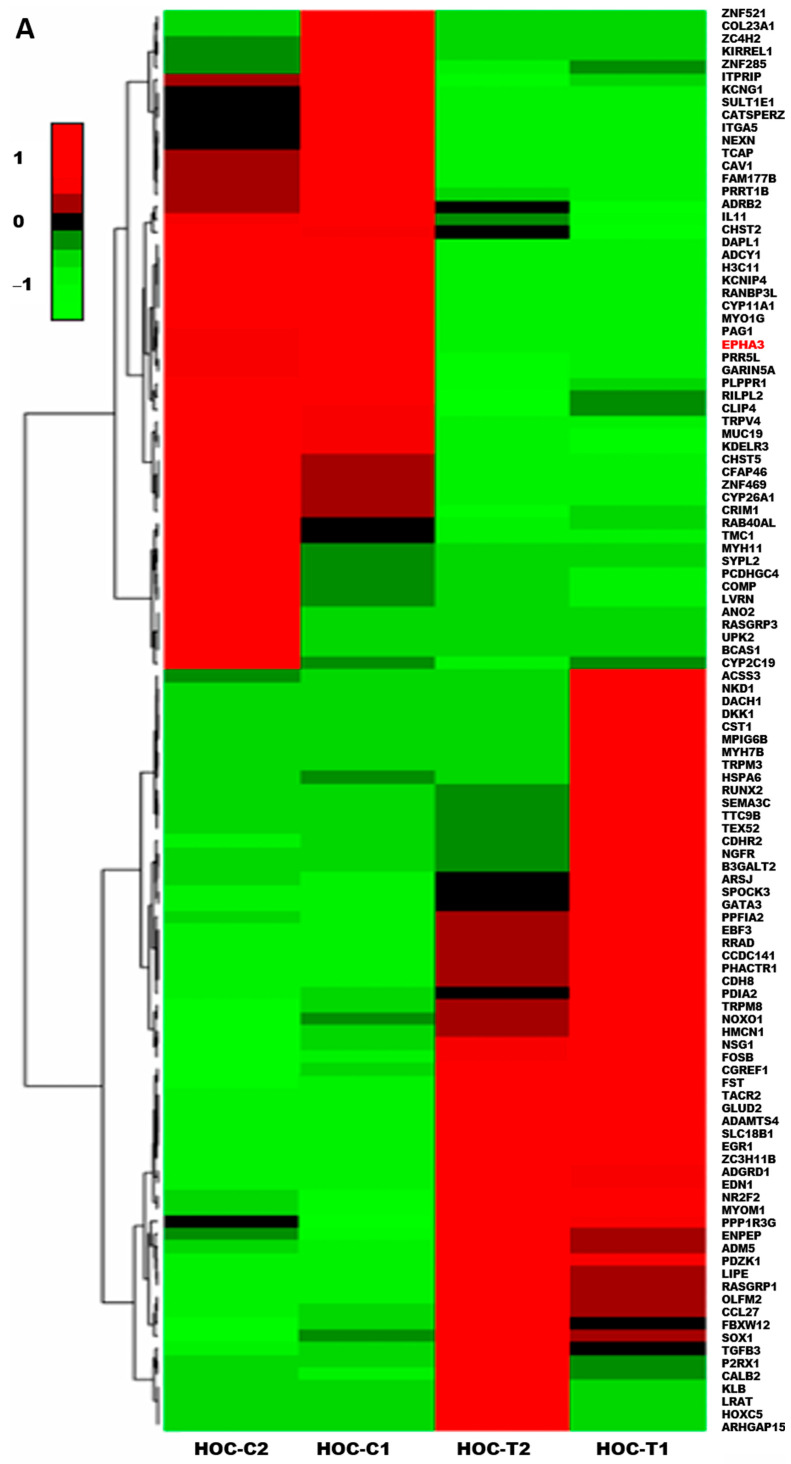

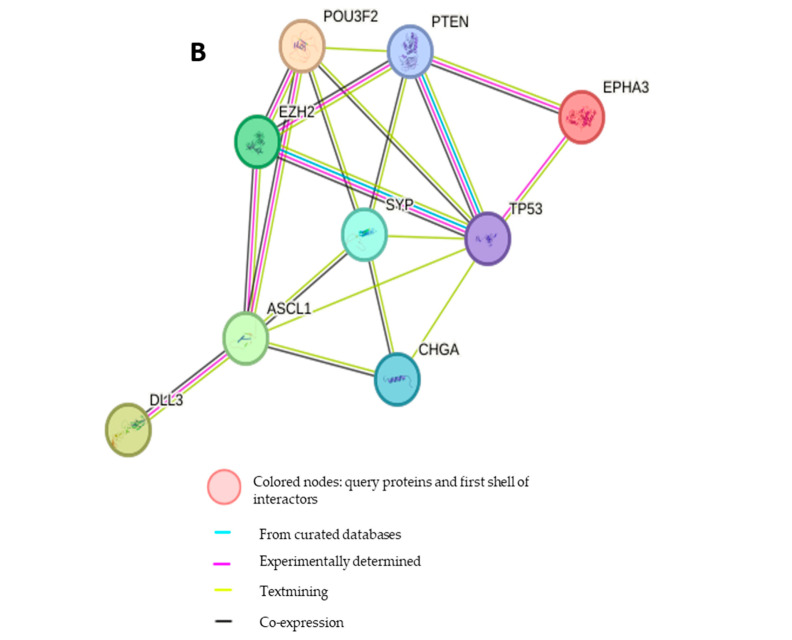
Treatment effects of HOC in the NCI-H660-Luc NEPC nude mouse xenograft model using RNA-Seq DEGs heatmap and PPI network analysis. (**A**) Heatmap RNA sequencing analysis comparing DEGs in placebo control versus HOC-treated NCI-H660-Luc tumors identified significantly differentially expressed genes (|log_2_FC| > 1.5, adjusted *p* < 0.05). The red text defines EPHA3 among the top downregulated genes. (**B**) STRING-based PPI network analysis depicting PPIs between EPHA3 and key NEPC markers EZH2, ASCL1, BRN2, DLL3, SYP, and CHGA, highlighting its critical role for NEPC pathogenesis.

Numerous studies reported the EPHA3 overexpression in several malignancies, including PCa, where it correlated with high aggression and invasive tumor potential [8,9]. Thus, to investigate the transcriptional impact of HOC daily oral treatment modalities on the NEPC, RNA sequencing was performed on NCI-H660-Luc primary tumors comparing HOC treatment effects on differentially expressed genes (DEGs) versus placebo control tumors (Supplementary Figure S2). This analysis identified a total of 121 DEGs, defined by a log_2_ fold-change threshold of >1.5 or <−1.5 and adjusted *p* < 0.05, including 67 upregulated genes and 54 downregulated genes. The heatmap of the DEGs comparing HOC with the placebo-treated NCI-H660-Luc tumors indicated the significant downregulation of EPHA3 uniquely observed following HOC treatment (log2 fold change: −17.82) (Figure 4A), whereas oleocanthal treatments showed a minimal change in the total EPHA3 expression (log2 fold change: −1.25).

### 3.5. Protein–Protein Interaction Network of EPHA3 with Key NEPC Markers

To further investigate the biological and molecular significance of EPHA3 in the NEPC context, a protein–protein interaction (PPI) network was generated using the STRING database, incorporating EPHA3 and canonical NEPC-related markers including EZH2, ASCL1, BRN2 (POU3F2), DLL3, SYP, and CHGA (Figure 4B). The network included EPHA3, along with neuroendocrine transcription factors EZH2 and ASCL1, and their upstream regulator BRN2 [17,18]. DLL3, a protein highly expressed in NEPC and known to regulate ASCL1, was also incorporated [12]. Furthermore, established NEPC markers such as synaptophysin (SYP) and chromogranin A (CHGA) were included to assess the broader network connectivity. The PPI analysis revealed significant interactions and straight interconnections among EPHA3, DLL3, BRN2, EZH2, ASCL1, SYP, and CHGA, indicating a coordinated molecular network that may be impacted by EPHA3 dysregulation in NEPC.

### 3.6. HOC Suppressed Tumor Recurrence Through Downregulation of EPHA3 and Disruption of Neuroendocrine Signaling Network

The HOC main anti-NEPC molecular pathway was suggested by the significant downregulation of the total EPHA3 level in the RNA-Seq results of treated collected NCI-H660-Luc primary tumors. The RTK EPHA3 is involved in cell adhesion, migration, and intercellular communication. Its downregulation could attenuate the tumor cells’ plasticity and suppress pro-tumorigenic signaling [5,6]. Previous studies have also implicated EPHA3 in angiogenesis and invasive pathways in various cancers, further supporting the hypothesis that HOC-mediated suppression of EPHA3 contributes to reduced tumor recurrence and aggressiveness observed in this study model [5,6]. The PPI network findings imply that HOC may disrupt a coordinated EPHA3-centered signaling module that connects with EZH2, ASCL1, and BRN2-regulated transcriptional programs. Downregulation of EPHA3 might therefore diminish the NED and interfere with PCa cells lineage plasticity, the key drivers of recurrence in t-NEPC. RNA-Seq results reveal that HOC might downregulate, disrupt EPHA3 interaction network with critical NEPC regulators such as DLL3, BRN2, EZH2, ASCL1, SYP, and CHGA. The distinct transcriptional and signaling consequences of EPHA3 suppression by HOC highlight its potential as a therapeutic molecular target in NEPC.

### 3.7. HOC Attenuates NEPC Pathogenesis Markers in NCI-H660-Luc Primary Tumor via Downregulation of EPHA3 and Associated Regulators

**Figure 5:**
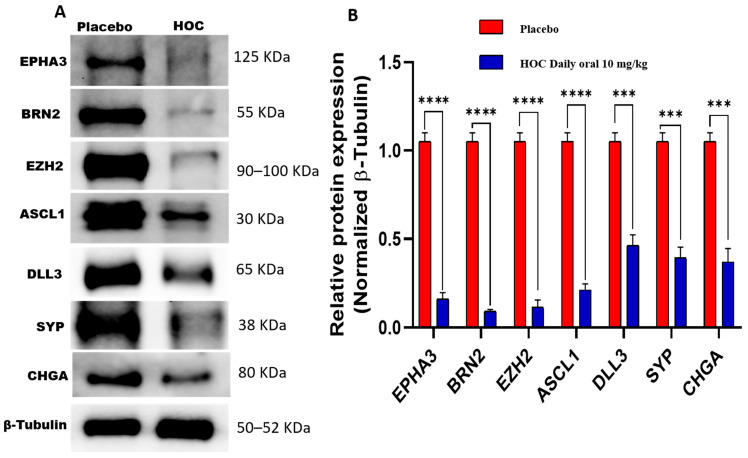
Western blotting assessment of the molecular effects of HOC treatments on NCI-H660-Luc primary tumors. (**A**) Western blot analysis illustrates the molecular effects of HOC treatments versus placebo on EPHA3, BRN2, EZH2, ASCL1, DLL3, SYP, and CHGA expression levels in collected NCI-H660-Luc primary tumors. (**B**) Corresponding bar graphs presentation of normalized optical density quantification for each protein. Densitometric quantification was based on triplicate blots, with band intensities normalized to β-tubulin as a loading control. Tumor lysates were obtained by pooling tissues from all tumors per group. Data are shown as mean ± SEM (*n* = 3). Statistical differences were determined using Student’s *t*-test, with *** *p* < 0.001 and **** *p* < 0.0001 indicating significance.

HOC treatments markedly decreased the protein expression of several NEPC key markers in NCI-H660-Luc primary tumors analyzed by Western blotting. Specifically, EPHA3 expression was notably reduced by 85% (Figure 5, Figures S3 and S4). BRN2, the NE transcription factor upstream of EZH2 and ASCL1, showed a 91.4% expression reduction. EZH2, a downstream substrate of EPHA3 key lysine methyltransferase, expression reduced by 89.5%. ASCL1, a factor influenced by BRN2, was downregulated by 80% in HOC-treated tumors. DLL3, which is downstream of ASCL1, exhibited a 56.2% reduction in expression by HOC treatments. Moreover, the standard NEPC markers CHGA and SYP were significantly decreased by 64.8% and 66%, respectively, in HOC-treated tumors (Figure 5). The observed downregulation of EPHA3, BRN2, EZH2, ASCL1, DLL3, SYP, and CHGA following HOC treatment in NCI-H660-Luc primary tumors suggests a broad impact on pathways involved in NEPC progression.

### 3.8. HOC Suppressed Neuroendocrine Phenotype in NCI-H660-Luc Recurrent Tumor via Downregulation of EPHA3 and Associated Regulators

**Figure 6:**
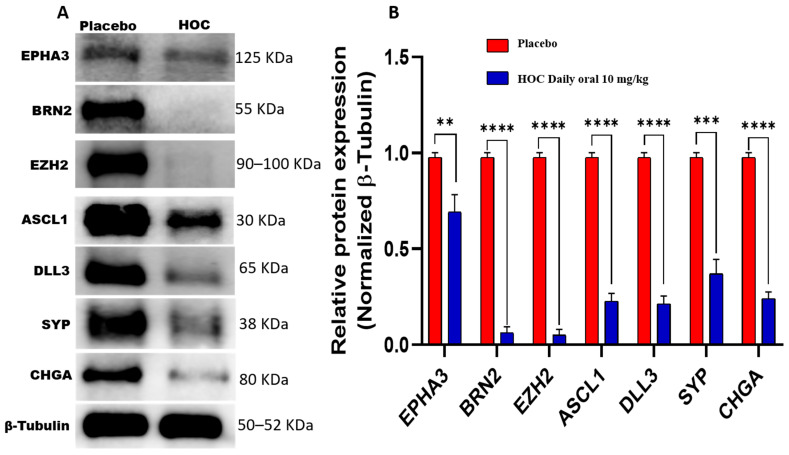
Western blotting assessment of the molecular effects of HOC on NCI-H660-Luc recurrent tumors. (**A**) Western blot analysis demonstrates the comparative effects of HOC versus placebo on EPHA3, BRN2, EZH2, ASCL1, DLL3, SYP, and CHGA expressions in NCI-H660-Luc recurrent tumors collected at the study end. (**B**) Corresponding bar graphs displaying normalized optical density values for each protein. Densitometric quantification was performed using triplicate blots, with protein intensities normalized to β-tubulin as a loading control. Tumor lysates were prepared by pooling tissues from two tumors in the control group and from one tumor in the HOC-treated group. Data are presented as mean ± SEM (*n* = 3). Statistical significance was determined using Student’s *t*-test, with ** *p* < 0.01, *** *p* < 0.001 and **** *p* < 0.0001 considered significant.

To elucidate the sustained molecular effects of HOC on NEPC during tumor recurrence, protein expression levels of EPHA3, BRN2, EZH2, ASCL1, DLL3, SYP, and CHGA were examined in NCI-H660-Luc recurrence tumors collected at the study end, which were compared with those observed in primary tumors. Western blot analyses demonstrated that HOC continued to exert significant inhibitory effects on EPHA3 network during tumor recurrence, though the magnitude of suppression varied among specific targets (Figure 6). In the recurrence tumors, HOC reduced EPHA3 expression by 31%. BRN2 showed a substantial reduction of 93.7%, while EZH2 expression was decreased by 95%. ASCL1 exhibited a 77% reduction in expression level, and its downstream target DLL3 was also significantly downregulated by 79%. Furthermore, the NEPC markers CHGA and SYP also experienced considerable 76% and 63% reductions, respectively (Figure 6).

Comparative molecular analysis of primary versus recurrence tumors reveals stage-specific differences in HOC-mediated modulation of the key neuroendocrine signaling proteins (Table 1). The reduction in EPHA3 expression was less pronounced in recurrent versus primary tumors (31% vs. 85%). Conversely, HOC-induced downregulation of DLL3 was more pronounced in recurrence tumors (79%) compared to progression (56.2%, Table 1). BRN2 remained profoundly suppressed by HOC across both stages (91.4% in progression versus 93.7% in recurrence). Moreover, the comparable inhibition of EZH2 (89.5% in progression versus 95% in recurrence) and ASCL1 (80% in progression versus 77% in recurrence) by HOC in both progression and recurrence phases and CHGA suppression increased from 64.8% in progression to 76% in recurrence, while SYP suppression levels were comparable (66% versus 63%).

## 4. Discussion

This study provides compelling evidence that HOC, a naturally-occurring secoiridoid phenolic derived from EVOO, exerts potent suppressive effects against NEPC both in vitro and in vivo. The results demonstrate that HOC effectively suppressed NEPC progression and recurrence through transcriptional and post-translational downregulation of EPHA3 and its interconnected oncogenic signaling network encompassing BRN2, EZH2, ASCL1, DLL3, SYP, and CHGA. The EPHA3-BRN2-EZH2-ASCL1-DLL3-SYP-CHGA axis constitutes a critical oncogenic network driving neuroendocrine transdifferentiation in advanced PCa. EPHA3 activation induces BRN2, which function together with the epigenetic lysine methyltransferase regulator EZH2, silencing the luminal lineage genes via H3K27 trimethylation, establishing a chromatin environment that permits neuroendocrine reprogramming. This facilitates upregulation of ASCL1, a master neuronal transcription factor that activates DLL3, reinforcing the neuroendocrine phenotypic markers SYP and CHGA expression, signifying NED. Importantly, this network also underlies therapy resistance, as the coordinated epigenetic and transcriptional reprogramming enables NEPC cells to resist AR-targeted therapies and standard treatments. These findings identify EPHA3 as a novel HOC molecular target in NEPC and highlight its potential as a nutraceutical therapeutic lead for controlling this highly PCa aggressive subtype that lacks current effective therapeutic interventions.

HOC exhibited a notable antiproliferative activity, particularly observed against advanced PCa subtypes represented by the mCRPC CWR-R1ca and the NEPC NCI-H660 cells. The improved comparative in vitro activity against NCI-H660 cells versus other tested cell lines such as LNCaP, PC-3, PC-3M, and DU-145 underscored its anti-NEPC potential. Thus, the NEPC NCI-H660-Luc cells represented a suitable model for in vivo investigation of NEPC in a nude mouse xenograft model, as it closely recapitulates the molecular and phenotypic characteristics of this aggressive subtype of PCa [37]. Moreover, while the non-adherent NCI-H660 cells display a slow in vitro proliferation rate, they demonstrate robust and effective tumor formation and progression when xenografted in immunodeficient mice, further supporting relevance as a valid preclinical model for evaluating HOC anti-NEPC therapeutic and molecular mechanistic responses [38].

The in vivo xenograft studies revealed that daily oral administration of HOC 10 mg/kg over 51 days substantially suppressed NEPC progression, reducing the NCI-H660-Luc tumor volume and weight by 85.5% and 79.1%, respectively, compared to the placebo control group. Complete tumor regression observed in one of the five HOC-treated mice (20%) further underscored a therapeutic potential against NEPC. Importantly, no significant body weight changes were observed, indicating excellent tolerability and absence of systemic toxicity. The applied 10 mg/kg mouse dose corresponds to a human equivalent dose (HED) of 0.81 mg/kg, based on the established interspecies allometric scaling factor (Km = 0.081 for mouse-to-human conversion) [39]. When this is extrapolated to a 70 kg average adult human weight, it translates to an estimated 56.7 mg of HOC per day. A good quality EVOO may contain up to 600 mg/L HOC, which corresponds to the intake of 94.5 mL/day of this quality EVOO. Such daily dosage is achievable within the practical range of daily EVOO consumption, emphasizing the translational potential of HOC as a feasible nutraceutical lead candidate for the management of NEPC. The significant anti-NEPC potency, excellent safety profile, and physiologically realistic human equivalent dose position HOC as a promising nutraceutical lead candidate for further translational consideration as NEPC management. The reduction in recurrence incidence highlights the potential of HOC to prevent or suppress the persister residual tumor cells regrowth, minimizing tumor relapse following surgical resection. Each 2.6 days of a mouse’s lifespan is approximately equivalent to one human year [40]; therefore, the 75-day study period in the mouse model will roughly be equivalent to 28.9 years of disease-free survival, reinforcing the translational significance of this study’s findings. A 14-day delay by HOC treatments in recurrence initiation translationally approximates 5.4 years of human life, suggesting that HOC may significantly extend progression-free survival periods in a clinical context. Such latency extension indicates that HOC could suppress early proliferative reactivation of residual tumor cells. The absence of distal tumor dissemination in HOC-treated animals suggests that HOC may not only suppress the locoregional recurrence but also impede the secondary metastatic seeding and distant recurrence. Collectively, these findings indicate that HOC treatment substantially suppressed NEPC recurrence, delayed recurrence onset, and aborted tumor vascularization without eliciting observable in vivo toxicity. Thus, HOC is a promising nutraceutical lead appropriate for the control of the aggressive NEPC progression and recurrence.

The RTK EPHA3 is critically implicated in tumor cells adhesion, migration, and intercellular communication. It is aberrantly dysregulated in several cancers, including PCa, where it correlates with aggressive phenotypes and metastasis potential [5,6,7,8,9]. The marked suppression of EPHA3 by HOC suggests interference with upstream signaling cues that drive NEPC pathogenesis. The neural-lineage transcription factor BRN2 is regulated by c-MYC and N-MYC and considered a master driver of NED in PCa by directly regulating EZH2 and ASCL1 expressions [17,18,19]. EZH2, the catalytic subunit of the PRC2 complex, promotes chromatin remodeling and represses tumor suppressor genes, contributing to lineage plasticity and therapy resistance [18,19]. The concurrent downregulation of BRN2 (91.4%) and EZH2 (89.5%) by HOC treatments underscores a potent suppression of this oncogenic transcriptional axis in NCI-H660 tumor cells. The key neurogenic transcription factor ASCL1 and its downstream effector DLL3 are hallmark regulators of neuroendocrine lineage commitment and are highly expressed in NEPC [13,41,42,43]. Similarly, the concurrent HOC treatment-induced reductions of the ASCL1 (80%) and DLL3 (56.2%) expression levels suggest impaired NED signaling. Likewise, the suppressed expression of the classical neuroendocrine markers CHGA (64.8%) and SYP (66%) further support the attenuation of the neuroendocrine phenotype by HOC treatment. These findings suggest that HOC could be a promising therapeutic lead by targeting multiple crucial proteins and thereby potentially mitigating the aggressive tumorigenicity of NEPC. The observed reduction in oncogenic culprit protein expression during the recurrence phase by HOC treatments in NCI-H660 cell xenograft model tumors underscores its therapeutic potential in managing recurrent NEPC.

Comparative molecular analysis suggests the differential effects of EPHA3 may play a more dominant role in NED or in the initial NEPC development stages or that there are compensatory mechanisms in recurrent tumors that might reduce HOC’s impact over extended exposure. DLL3, a downstream effector of ASCL1 and a Notch signaling inhibitor is highly expressed in NEPC and small cell cancers [13,42,43]. The stronger HOC treatments’ suppression of DLL3 during recurrence may reflect enhanced sensitivity of downstream signaling components once the upstream regulators BRN2, EZH2, and ASCL1 remain chronically repressed. This extended reduction in DLL3 expression suggests the effective sustained pressure against NE-specific Notch signaling circuitry by HOC treatments [42]. Comparative suppression of BRN2 by HOC across both stages indicating a durable inhibitory effect for this critical neural-lineage transcription factor. The sustained downregulation of BRN2 by HOC underscores its long-term capacity to repress transcriptional programs driving lineage plasticity and therapy resistance in NEPC. Moreover, the comparable inhibition of EZH2 and ASCL1 by HOC treatments both in progression and recurrence models demonstrate effective oncogenic transcription repression maintenance and consistent suppression of neuroendocrine lineage-determining transcription factors, which contributed to delayed or weakened tumor relapse. These results indicate that HOC effectively maintained a long-term inhibition of NE marker expression, reinforcing its suppressive effects for NEPC pathogenesis during both progression and recurrence phases. HOC targets a broad spectrum of oncogenic pathways essential for both primary tumor progression and recurrence. This multi-target action could be highly beneficial in overcoming the challenges associated with recurrent NEPC, a highly aggressive and therapy-resistant subtype of PCa [13,42,43].

The observed oral therapeutic effects and preliminary safety profile of HOC shown in this study were consistent with literature [44,45,46,47,48]. HOC displayed a moderate intestinal absorption and underwent extensive first-pass metabolism that limited its systemic exposure. In a rat single-pass intestinal perfusion model, HOC exhibited ~49% absorption with a high apparent permeability. However, rapid enterocyte metabolism generated the HOC parent alcohol HT and multiple oxidized metabolites that predominated in systemic plasma over intact HOC [44]. The tissue distribution studies detected unmetabolized HOC in stomach, small intestine, liver, plasma, and notably heart [45]. Whereas multiple phase I/II metabolites were widely distributed across other organs, indicating extensive in vivo biotransformation [45]. Pharmacokinetic studies suggested rapid intestinal and liver metabolism, which limited HOC bioavailability [46]. Gut microbiota metabolism of HOC produced HT and other oxidized metabolites [46]. Accordingly, de-phenolized EVOO was used as the HOC delivery vehicle in this study to enhance its metabolic stability and limit rapid gastrointestinal degradation following oral administration. In a high-fat diet–fed mouse model, histological analyses demonstrated that oral 20 mg/kg HOC notably reduced adipocyte hypertrophy and inflammatory immune cells infiltration in adipose tissues. HOC downregulated the key adipogenic and lipogenic markers, including peroxisome proliferator-activated receptor gamma and fatty acid synthase and restored insulin-sensitive muscle/fat glucose transporter GLUT-4 expression in skeletal muscle and adipose tissue, indicating improved insulin sensitivity and metabolic homeostasis [47]. HOC prevented body weight increase, liver enlargement, and plasma metabolic glucose and cholesterol disturbances caused by high-fat diets [48].

## 5. Conclusions

The daily oral administration of HOC at 10 mg/kg in the NEPC NCI-H660 xenograft animal model effectively targeted the EPHA3–BRN2–EZH2–ASCL1–DLL3–SYP–CHGA signaling network, achieved 20% complete tumor regression and effectively suppressed NEPC progression and locoregional and distant recurrences. This highlights its potent ability to modulate a critical oncogenic network driving NEPC lineage plasticity, epigenetic reprogramming, and therapeutic resistance. These findings establish HOC as a novel nutraceutical lead intervention useful for controlling NEPC.

## Figures and Tables

**Table 1:** Comparison of the NEPC oncogenic driver expression levels reduction between HOC-treated primary and recurrent NCI-H660-Luc tumor based on Western blot densitometric analyses.

Protein Name	Primary Tumor % Reduction	Recurrence Tumor % Reduction
EPHA3	85	31
BRN2	91.4	93.7
EZH2	89.5	95
ASCL1	80	77
DLL3	56.2	79
CHGA	64.8	76
SYP	66	63

## Data Availability

All data used to support the findings of this study made available in this publication as figures, tables, or Supplementary Materials. RNA-Seq data available upon request.

## Supplementary Materials

The following supporting information can be downloaded at: https://www.mdpi.com/article/10.3390/cancers18010026/s1, Supplementary Figure S1. Exploration of collected organs from experimental animals for bioluminescent distant recurrence/micro-metastasis tumor patches; Supplementary Figure S2. Volcano plots illustrating differentially expressed genes (DEGs) between placebo control and HOC-treated NCI-H660-Luc tumor tissues; Supplementary Figures S3 and S4. Raw Western blot gels for all proteins assessed in the study.
