# Supplementary material for: The Olive Phenolic S-(-)-Hydroxyoleocanthal Attenuates Neuroendocrine Prostate Cancer via Modulation of EPHA3-Centered Oncogenic Network
Source: Cancers (Basel). 2025 Dec 21;18(1):26. doi: 10.3390/cancers18010026 (PMC12785029; doi:10.3390/cancers18010026)
Supplement: Supplementary file 1 [file cancers-18-00026-s001.zip › cancers-4015198-supplementary.pdf]

cancers

Supplementary Materials

The Olive Phenolic *S*-(-)-Hydroxyoleocanthal Attenuates Neuroendocrine Prostate Cancer via Modulation of EPHA3-Centered Oncogenic Network

Md Towhidul Islam Tarun [1], Hassan Y. Ebrahim [1,2] and Khalid A. El Sayed [1,*]

[1] School of Basic Pharmaceutical and Toxicological Sciences, College of Pharmacy, University of Louisiana at Monroe, 1800 Bienville Drive, Monroe, LA 71201, USA; tarunmt@warhawks.ulm.edu (M.T.I.T.); hebrahim@ulm.vcom.edu (H.Y.E.)
[2] Department of Biomedical Sciences, Discipline of Pharmacology, Edward Via College of Osteopathic Medicine, Monroe, LA 71201, USA
* Correspondence: elsayed@ulm.edu; Tel.: +1-318-342-1725

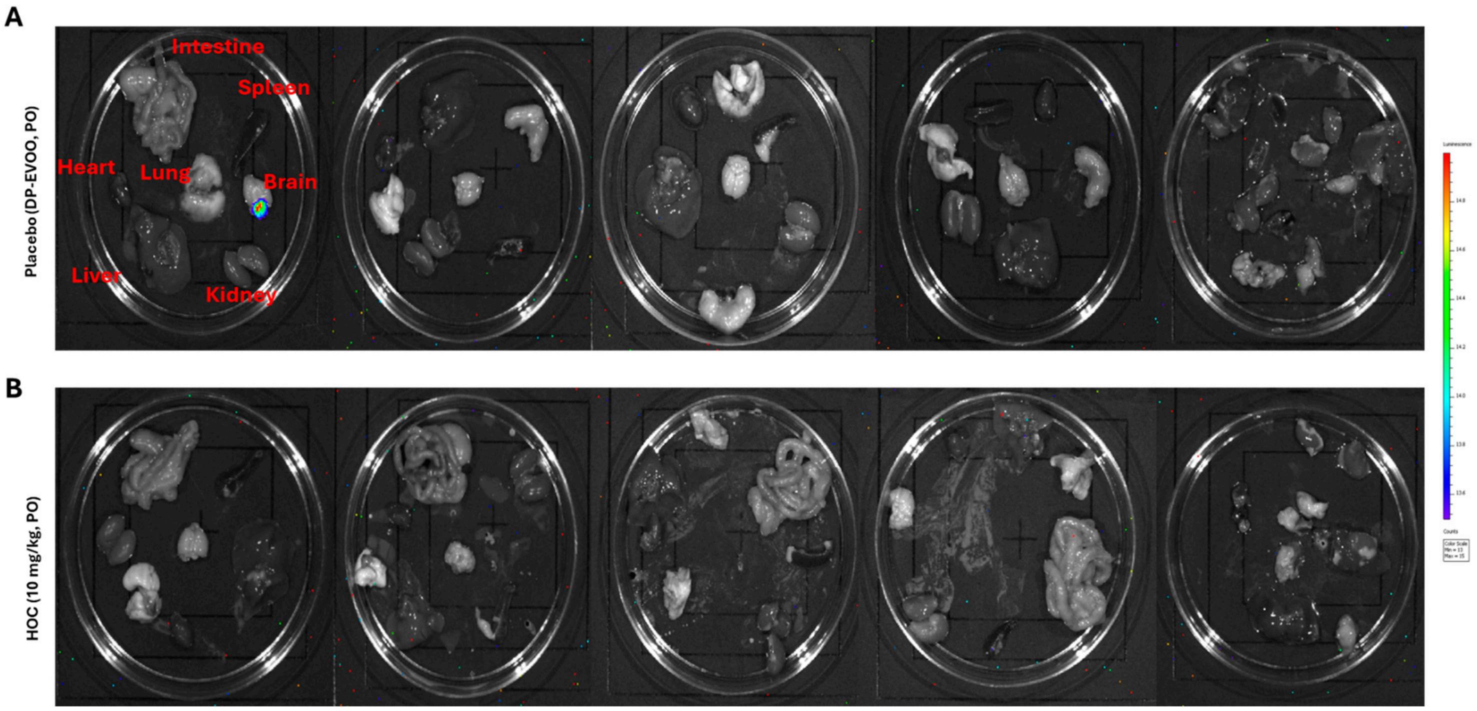

Supplementary Figure S1. Exploration of collected organs from experimental animals for bioluminescent distant recurrence/micro-metastasis tumor patches. Distant recurrence was observed in 1 out of 5 mice in the placebo control group (brain, top left image) and in 0 out of 5 mice in the HOC-treated group, indicating the absence of metastatic recurrence following HOC treatments.

https://doi.org/10.3390/cancers18010026

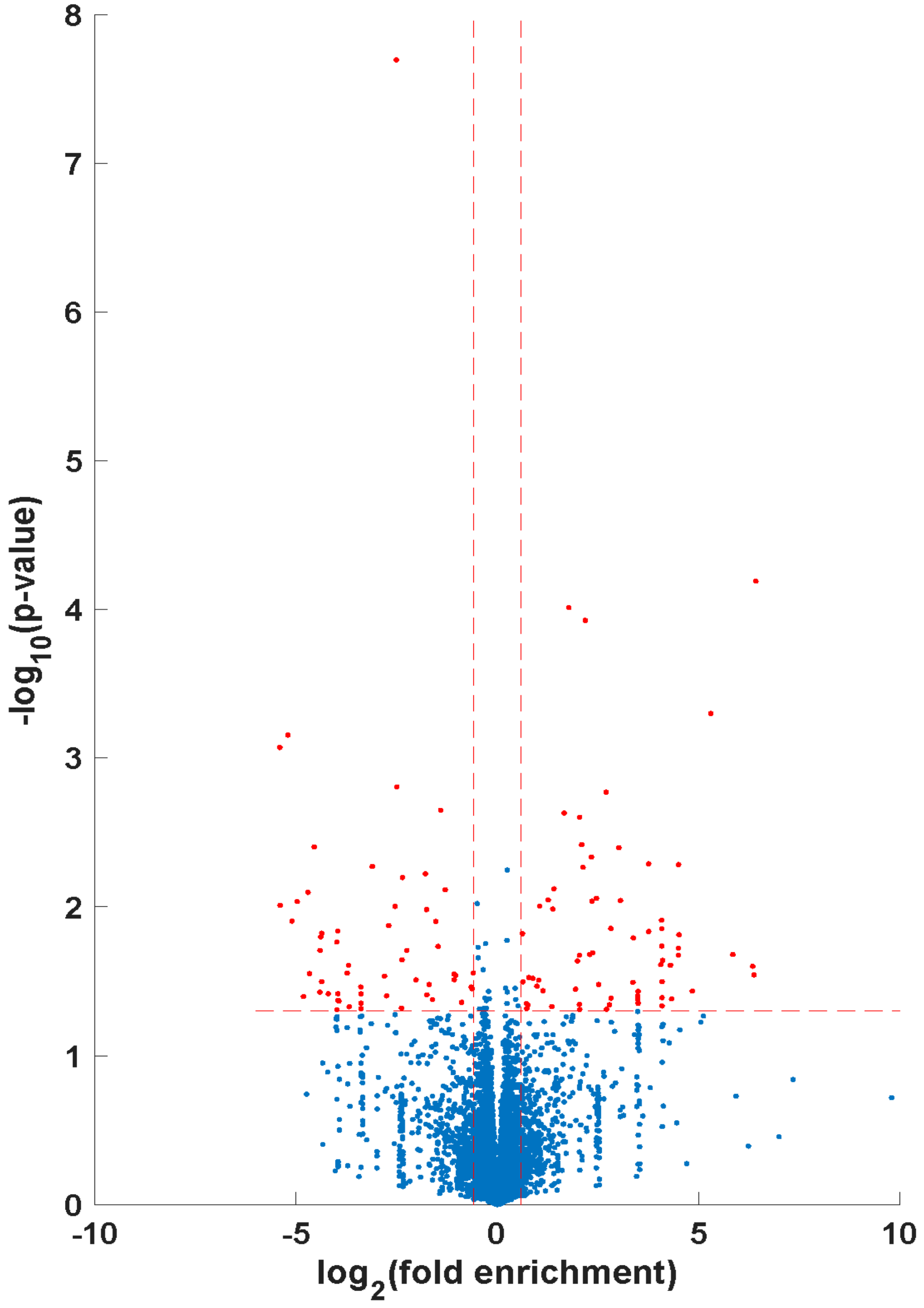

Supplementary Figure S2. Volcano plots illustrating differentially expressed genes (DEGs) between placebo control and HOC-treated NCI-H660-Luc tumor tissues. Significantly upregulated and downregulated DEGs are highlighted in red, while non-significant genes are shown in blue. The vertical red lines indicate $\log_2$ fold-change thresholds of −1.5 and 1.5, and the horizontal red line represents an adjusted *p*-value cutoff of 0.05.

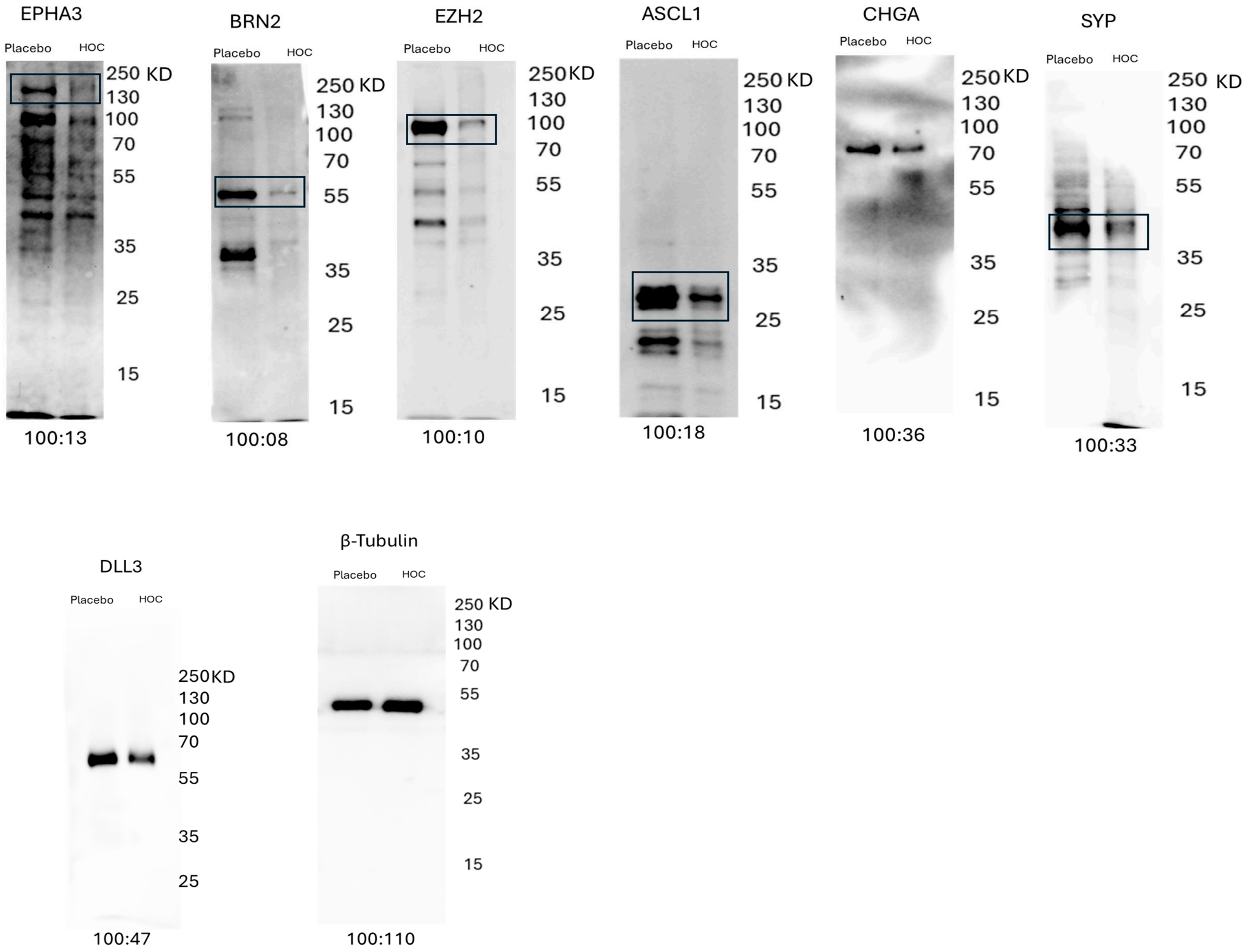

Supplementary Figure S3. Raw Western blot images demonstrating the impact of HOC treatment on key oncogenic and neuroendocrine markers (EPHA3, BRN2, EZH2, ASCL1, DLL3, SYP, and CHGA) in NCI-H660-Luc tumor tissues collected after the progression phase. β-Tubulin was used as the internal loading control.

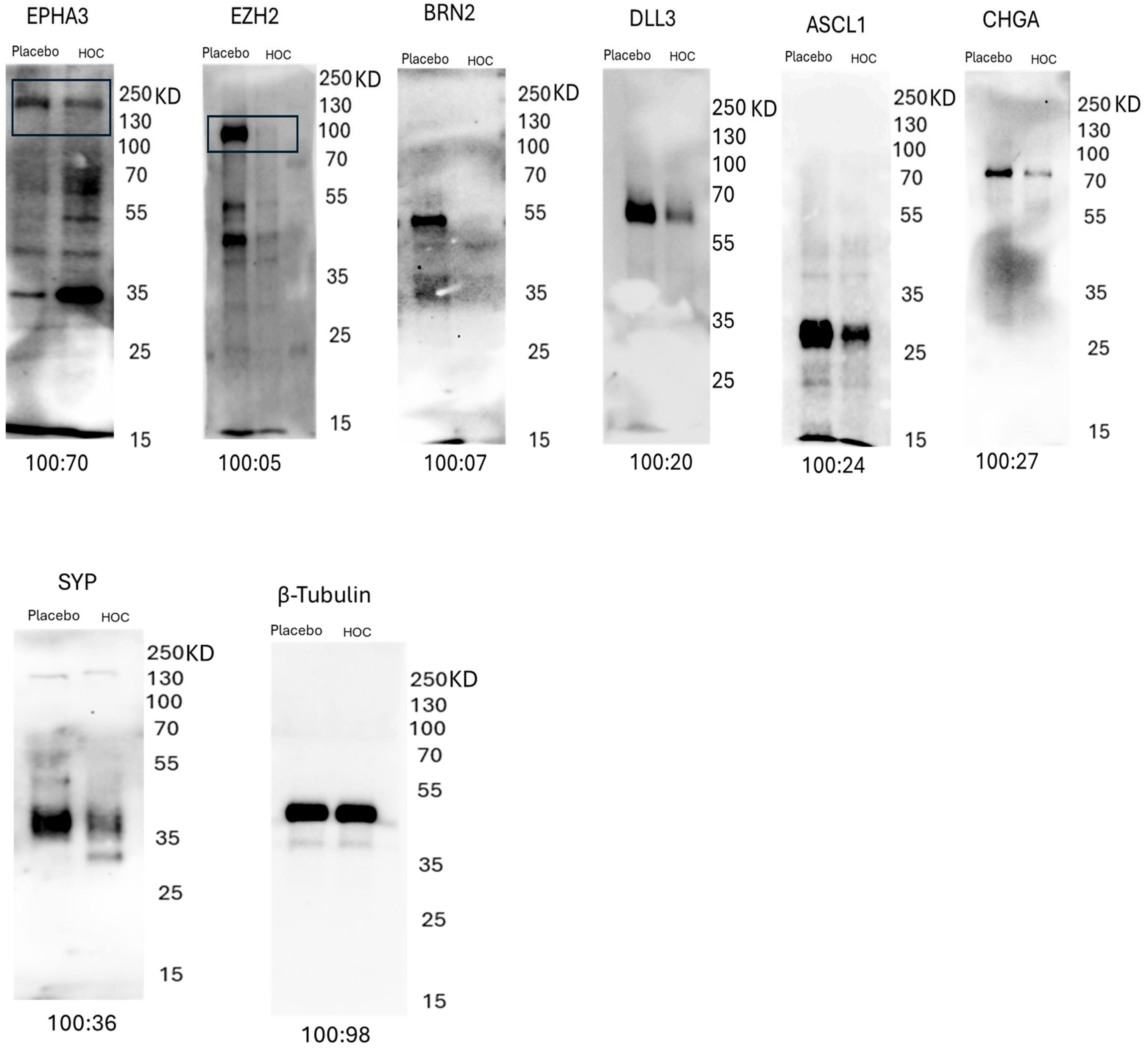

Supplementary Figure S4. Raw Western blot images illustrating the effect of HOC treatment on the expression of EPHA3, BRN2, EZH2, ASCL1, DLL3, SYP, and CHGA in NCI-H660-Luc tumor tissues collected after the recurrence phase. β-Tubulin served as the loading control.

https://doi.org/10.3390/cancers18010026
